# Established risk factors for type 2 diabetes in adults with depression: implications for endocrine practice

**DOI:** 10.1186/s12902-026-02342-7

**Published:** 2026-06-09

**Authors:** Jordan Zuber, Michael L. Thomas, Murray B. Stein, Alejandro D. Meruelo

**Affiliations:** 1 Department of Psychiatry, University of California, San Diego, USA; 2 Department of Psychology, Colorado State University, Fort Collins, USA; 3 Department of Psychiatry, VA San Diego Healthcare System, San Diego, USA

**Keywords:** Type 2 diabetes mellitus, Depression, Metabolic risk factors, Obesity, Socioeconomic status, Endocrine epidemiology, All of us research program

## Abstract

**Background:**

Type 2 diabetes mellitus (T2DM) shares multiple modifiable and non-modifiable risk factors across populations, yet it is unclear whether these risk profiles differ in adults with depression—a group with elevated baseline metabolic risk.

**Objective:**

To identify clinical, behavioral, and sociodemographic predictors of incident T2DM among adults with depression and determine their implications for endocrine prevention and screening strategies.

**Methods:**

We conducted a retrospective cohort analysis of 40,585 adults with a documented depression diagnosis but no prior T2DM in the All of Us Research Program (median follow-up = 8.15 years). Incident T2DM was defined using electronic health record–based diagnosis codes. Cox proportional hazards models estimated adjusted hazard ratios (HRs) for T2DM as a function of gender, race, ethnicity, age, body mass index (BMI per 10 kg/m²), and household income. Sensitivity analyses incorporated cigarette smoking and multiple imputation for BMI.

**Results:**

Higher BMI (HR = 1.76 per 10 kg/m²; 95% CI: 1.69–1.83), older age (HR = 1.03 per year; 95% CI: 1.027–1.032), male gender (HR = 1.38; 95% CI: 1.27–1.49), Black race (HR = 1.79; 95% CI: 1.64–1.95), and non-Hispanic ethnicity (HR = 1.37; 95% CI: 1.10–1.71) were independently associated with greater T2DM risk, while higher household income was protective (HR = 0.47 for >$150k vs. <$35k; 95% CI: 0.39–0.58), demonstrating a clear socioeconomic gradient. Associations were robust to smoking adjustment and multiple imputation, and were directionally consistent with patterns reported in prior general-population studies.

**Conclusions:**

Traditional demographic, socioeconomic, and clinical risk factors were associated with incident T2DM among adults with depression, supporting attention to established diabetes risk factors in psychiatric and endocrine prevention settings. Aggressive implementation of weight management, risk stratification, and early screening—particularly among socioeconomically disadvantaged patients—may reduce the burden of T2DM in psychiatric populations.

**Clinical trial number:**

Not applicable.

**Supplementary Information:**

The online version contains supplementary material available at 10.1186/s12902-026-02342-7.

## Introduction

Type 2 diabetes mellitus (T2DM) and major depressive disorder are two of the most prevalent and burdensome chronic health conditions globally, each associated with substantial morbidity, impaired quality of life, and increased mortality [1, 2]. Epidemiologic studies have consistently shown that depression is not only highly comorbid with T2DM but also a prospective risk factor for its onset [3]. Proposed mechanisms linking depression to T2DM include dysregulation of the hypothalamic-pituitary-adrenal (HPA) axis, increased inflammation, alterations in appetite-regulating hormones, poor sleep, and adverse health behaviors such as physical inactivity, tobacco use, and poor diet [4, 5]. Cigarette smoking, in particular, is more prevalent among individuals with depression and is independently associated with insulin resistance and increased T2DM risk [6, 7]. Antidepressant medications may further contribute to metabolic dysregulation, particularly through weight gain and insulin resistance [8]. As such, depression has been hypothesized to alter the trajectory of metabolic health and diabetes onset [9].

Sociodemographic factors are also well-established determinants of T2DM in the general population. Low socioeconomic status, particularly low household income, is robustly linked to higher diabetes risk through mechanisms involving nutrition, exercise opportunities, chronic stress exposure, and reduced access to preventive healthcare [10, 11]. Structural racism and social inequities contribute to persistently higher T2DM prevalence among Black and Hispanic individuals in the United States [12]. These social determinants likely interact with depression, potentially compounding metabolic vulnerability [13].

While the influence of BMI, age, income, race, and gender on T2DM risk is well documented in general population studies [14, 15], it remains unclear whether these associations exhibit similar patterns among adults with depression. Because depression independently increases diabetes risk, it is plausible that traditional risk factors could exert stronger, weaker, or qualitatively different effects in psychiatric populations. Moreover, modifiable behavioral risk factors such as cigarette smoking—more prevalent among individuals with depression and independently associated with insulin resistance and T2DM risk [6]—may further shape metabolic vulnerability within this group. If traditional and behavioral risk factors operate differently in depressed adults, this would have implications for risk stratification, screening thresholds, and prevention strategies within mental health care settings. Conversely, if risk patterns mirror those observed in the general population after accounting for key behavioral exposures, existing diabetes risk prediction and prevention frameworks may be directly applicable to individuals with depression. Despite the clinical importance of this distinction, few large-scale studies have directly evaluated whether established metabolic risk gradients—including behavioral contributors such as smoking [16]—are preserved in depressed adults, particularly in diverse cohorts with linked clinical and behavioral data.

To address this gap, we used data from the All of Us Research Program—a large, diverse cohort with electronic health records and survey data—to examine clinical, behavioral, and sociodemographic predictors of incident T2DM among adults with depression. Although the cohort is not nationally representative and reflects a broad convenience sample [17], its scale and diversity provide an opportunity to examine risk factor patterns within a well-characterized population. Our goal was to evaluate whether these predictors align with well-established risk factors from general population studies or whether distinct patterns emerge in depressed individuals. We hypothesized that higher BMI, older age, male gender, Black race, and lower household income would be associated with increased risk of T2DM after depression diagnosis and that the overall risk factor profile would largely mirror known patterns in non-depressed populations.

## Methods

### Study population

**Fig. 1:**
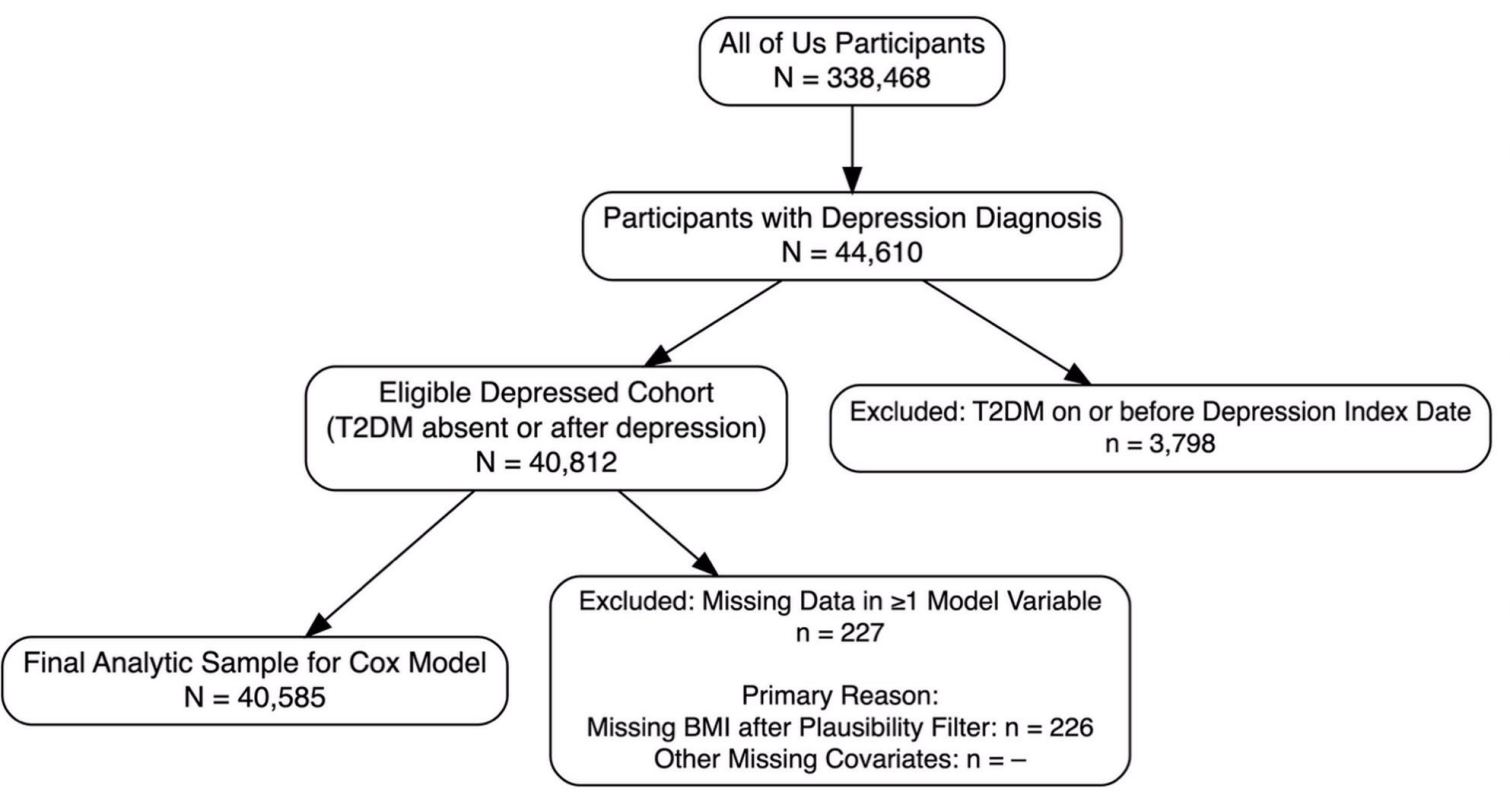
Participant flow diagram. Flow diagram of participant selection for the depression-to-incident T2DM cohort analysis in the All of Us Research Program. Participants with prevalent T2DM at or before the depression index date were excluded. Variables with counts ≤ 20 are masked (“-”) per All of Us policy

We conducted a retrospective cohort study using data from the All of Us Research Program Registered Tier dataset (version 8) [18], which includes electronic health records (EHRs), survey responses, and physical measurements from a diverse cohort of U.S. adults. Participants were eligible for inclusion if they had a documented diagnosis of depression (based on SNOMED codes in EHRs), were at least 18 years of age at the time of diagnosis, and had at least one year of follow-up data. Individuals with a pre-existing diagnosis of T2DM at or before the date of depression diagnosis were excluded. The final analytic sample included 40,585 participants (see Fig. 1 for participant flow diagram). Baseline sociodemographic characteristics are presented in Table 1; a comparison with nationally representative, survey-weighted estimates of U.S. adults with depression from the 2024 National Health Interview Survey [19] is provided in Supplementary Table 1. This design allowed us to evaluate whether established risk factors for T2DM observed in general population studies also predict diabetes onset in adults with depression.

**Table 1:** Participant demographics and clinical characteristics by incident T2DM status following depression diagnosis (*N* = 40,585)

Characteristic	Overall	No T2DM	Incident T2DM	*p*-value	SMD
**Sample Size**	40,585	37,303	3,282		
**Gender**,** n (%)**				< 0.001	0.101
Female	30,315 (74.7%)	27,979 (75.0%)	2,336 (71.2%)		
Male	9,386 (23.1%)	8,499 (22.8%)	887 (27.0%)		
Other/Unknown	884 (2.2%)	825 (2.2%)	59 (1.8%)		
**Race**,** n (%)**				< 0.001	0.298
White	22,685 (55.9%)	21,169 (56.7%)	1,516 (46.2%)		
Black	6,744 (16.6%)	5,852 (15.7%)	892 (27.2%)		
Asian	537 (1.3%)	512 (1.4%)	25 (0.8%)		
Other	3,484 (8.6%)	3,217 (8.6%)	267 (8.1%)		
Unknown	7,135 (17.6%)	6,553 (17.6%)	582 (17.7%)		
**Ethnicity**,** n (%)**				0.031	0.049
Hispanic or Latino	7,771 (19.1%)	7,196 (19.3%)	575 (17.5%)		
Not Hispanic or Latino	31,644 (78.0%)	29,042 (77.9%)	2,602 (79.3%)		
Other/Unknown	1,170 (2.9%)	1,065 (2.9%)	105 (3.2%)		
**Income**,** n (%)**				< 0.001	0.304
<$35k	15,148 (37.3%)	13,683 (36.7%)	1,465 (44.6%)		
$35k–50k	3,542 (8.7%)	3,243 (8.7%)	299 (9.1%)		
$50k–75k	4,371 (10.8%)	4,024 (10.8%)	347 (10.6%)		
$75k–100k	2,999 (7.4%)	2,838 (7.6%)	161 (4.9%)		
$100k–150k	3,540 (8.7%)	3,373 (9.0%)	167 (5.1%)		
>$150k	3,098 (7.6%)	2,985 (8.0%)	113 (3.4%)		
Unknown	7,887 (19.4%)	7,157 (19.2%)	730 (22.2%)		
**Age (years)**,** mean (SD)**	47.08 (16.23)	46.70 (16.44)	51.41 (12.84)	< 0.001	0.319
**BMI (per 10 kg/m²)**,** mean (SD)**	3.08 (0.79)	3.05 (0.78)	3.45 (0.79)	< 0.001	0.515

Demographic and clinical characteristics of adults with a diagnosis of depression, stratified by incident type 2 diabetes mellitus (T2DM) status. Values are presented as mean (SD) for continuous variables and count (percentage) for categorical variables. BMI is scaled per 10 kg/m². Standardized mean differences (SMDs) indicate effect size; values > 0.1 suggest meaningful imbalance. Group comparisons used chi-squared tests for categorical variables and t-tests for continuous variables. One observation was excluded due to missingness

### Outcome definition

The primary outcome was incident T2DM, defined using standardized concept identifiers consistent with clinical guidelines. Incident cases were identified from EHR condition records using SNOMED CT and OMOP concept IDs corresponding to type 2 diabetes, with diagnosis dates occurring after the index depression diagnosis (see Supplementary Appendix A for depression code list and Supplementary Appendix B for T2DM code list).

### Time at risk

Time at risk was calculated as the number of days from the depression diagnosis date to the earliest of the following events: first T2DM diagnosis, death, or last available follow-up date. Time was converted to years by dividing by 365.25.

### Covariates

Sociodemographic covariates included self-reported gender (Female, Male, Other/Unknown), race (White, Black, Asian, Other, Unknown), and ethnicity (Hispanic or Latino, Not Hispanic or Latino, Other/Unknown), collected from baseline surveys. Age at depression diagnosis was computed as the difference between date of birth and the date of first recorded depression diagnosis. Body mass index (BMI) was obtained from physical measurement records and averaged across available entries per participant. Implausible BMI values (< 10 or > 60 kg/m²) were excluded, and BMI was rescaled to reflect change per 10 kg/m² (bmi10) for model interpretability. BMI was modeled continuously to preserve information and avoid loss of statistical power associated with arbitrary categorization. We retained the continuous specification because the primary goal was to estimate the risk associated with increasing adiposity across the cohort.

Annual income was derived from responses to the survey question on total household income before taxes. Responses were grouped into seven categories: “<$35k”, “$35k–50k”, “$50k–75k”, “$75k–100k”, “$100k–150k”, “>$150k”, and “Unknown” (which included “Prefer Not to Answer” and skipped responses).

Smoking status was defined using self-reported endorsement of having smoked at least 100 cigarettes in one’s lifetime, a measure administered to the vast majority of participants. Smoking data were available for 40,812 individuals (99.6% of the analytic cohort), with minimal missingness (2.7%). Smoking was incorporated as an ever-smoker variable in a sensitivity analysis to evaluate the robustness of the primary associations.

### Handling of missing data

We excluded participants with missing data on any model covariates, including gender, race, ethnicity, age, BMI, and income category. Missing values were most common in the income variable, and participants who declined to report income or skipped the question were categorized as “Unknown” and retained in the analysis. Implausible or extreme BMI values were coded as missing prior to exclusion. The final dataset used for modeling included only complete cases and no imputation was performed.

### Sensitivity analyses: smoking and missing data

To evaluate the robustness of the primary model to adjustment for smoking, we conducted a sensitivity analysis including smoking as a covariate. Smoking was defined as endorsement of having smoked at least 100 cigarettes in one’s lifetime (ever-smoker vs. never-smoker), a standard epidemiologic definition that avoids skip-pattern bias associated with cigarettes-per-day items. Smoking was evaluated as a sensitivity covariate rather than included in the primary model because the available ever-smoking measure did not capture smoking intensity, duration, recency, or temporality relative to depression diagnosis and therefore may incompletely characterize tobacco-related metabolic risk. Accordingly, smoking was incorporated to evaluate the robustness of the primary associations rather than as a core prespecified predictor in the primary model. This binary ever-smoking variable was merged into the analysis dataset and incorporated into a multivariable Cox proportional hazards model alongside all primary covariates. Results from this extended model were compared with the base model to assess whether associations with gender, race, income, and BMI were meaningfully altered by inclusion of smoking.

To evaluate the potential impact of missing data on our findings, we also conducted a complete case sensitivity analysis. Participants with and without complete covariate data were compared across sociodemographic characteristics using chi-squared tests for categorical variables and t-tests for continuous variables. Standardized mean differences (SMDs) were used to assess imbalance, with values > 0.1 considered potentially meaningful. Differences in the distribution of race, income, and BMI were noted between complete and incomplete cases.

To explore patterns of missingness, we conducted Little’s test for missing completely at random (MCAR) using the MissMech package in R [20]. The test was applied to a random subsample of 2,000 participants and included all primary modeling variables: time at risk, event status, gender, race, ethnicity, age, BMI (per 10 kg/m²), and income category. Although the Hawkins test indicated deviations from multivariate normality, the non-parametric component of Little’s test—which is robust to such violations—yielded a non-significant result (*p* = 0.32). We therefore found no statistical evidence to reject the MCAR assumption in this subsample. However, because MCAR cannot be definitively established, missingness may still be consistent with a missing at random (MAR) mechanism.

Accordingly, we implemented multiple imputation using predictive mean matching to mitigate potential bias associated with incomplete data and to evaluate the robustness of model estimates under the assumption that data were MAR. Imputation was conducted using the mice package in R with predictive mean matching for continuous variables (e.g., BMI) and polytomous logistic regression for categorical variables (e.g., income category). Five imputed datasets (m = 5) were generated using all modeling variables as predictors. A Cox proportional hazards model was fit separately within each imputed dataset and then pooled using Rubin’s rules. Hazard ratios (HRs), standard errors, and 95% confidence intervals (CIs) from the pooled analysis were compared to the complete-case results to evaluate consistency. Visual comparison was provided via a forest plot of pooled estimates and full regression output was reported.

### Statistical analysis

Cox proportional hazards regression was used to estimate adjusted hazard ratios (HRs) and 95% confidence intervals (CIs) for incident T2DM. The primary model included gender, race, ethnicity, age (per year), BMI (per 10 kg/m²), and income category. Reference categories were Female for gender, White for race, Hispanic or Latino for ethnicity, and <$35k for income. Proportional hazards assumptions were evaluated using Schoenfeld residuals. We additionally tested interaction terms between BMI and gender as well as BMI and race, and conducted stratified models to explore subgroup-specific associations. Model discrimination was assessed using the concordance statistic (C-index).

All analyses were conducted in R (version 4.4.1) [21], using the survival [22], tableone [23], broom [24], and tidyverse [25] packages. Statistical significance was set at two-sided *p* < 0.05. Given the large sample size, most estimates were statistically significant; therefore, we interpreted effect sizes with reference to HR magnitude: HRs near 1.0 were considered negligible, HRs of 1.2–1.5 as small, 1.5–2.0 as moderate, and > 2.0 as large, in line with conventional epidemiologic interpretations [26].

## Results

We conducted a Cox proportional hazards analysis to evaluate predictors of incident type 2 diabetes mellitus (T2DM) following a diagnosis of depression among 40,585 adults in the All of Us cohort, adjusting for gender, race/ethnicity, age, body mass index (BMI), and other covariates. Participants with pre-existing T2DM were excluded, and follow-up time was calculated from the date of depression diagnosis until either T2DM onset or censoring. Mean follow-up was 8.97 years (SD = 4.83; median = 8.15 years), corresponding to 364,030 person-years of observation.

Baseline characteristics are summarized in Table 1. Participants who developed incident T2DM were older on average than those who did not develop T2DM and had higher mean BMI. Incident T2DM was also more common among male participants, Black participants, and those in the lowest household income category, with the largest standardized mean differences observed for BMI, age, race, and income.

### Main effects (Table 2; Fig. 2)

In the fully adjusted model, higher BMI was strongly associated with increased risk of incident T2DM (HR = 1.76 per 10 kg/m², 95% CI: 1.69–1.83, *p* < 0.001), representing a moderate-to-large effect. Older age was also independently associated with greater risk (HR = 1.03 per year, 95% CI: 1.027–1.032, *p* < 0.001), though the magnitude of this effect was small per unit increase.

Compared to White participants, those identifying as Black had substantially higher risk of T2DM (HR = 1.79, 95% CI: 1.64–1.95, *p* < 0.001), indicating a large effect. Participants classified as Other race (HR = 1.25, 95% CI: 1.09–1.44, *p* = 0.002) and Unknown race (HR = 1.74, 95% CI: 1.38–2.20, *p* < 0.001) also showed elevated risk, consistent with small-to-moderate and large effects, respectively, while risk did not significantly differ for Asian participants. Gender also played a role: males exhibited higher risk than females (HR = 1.38, 95% CI: 1.27–1.49, *p* < 0.001), reflecting a small-to-moderate effect, whereas Other/Unknown gender was not associated with risk. Ethnicity was significantly associated with T2DM risk, with participants identifying as Not Hispanic or Latino demonstrating higher risk compared to Hispanic participants (HR = 1.37, 95% CI: 1.10–1.71, *p* = 0.006), while Other/Unknown ethnicity was not significant.

Participants in higher income categories demonstrated a graded reduction in T2DM risk. Compared to those earning less than $35k, individuals earning $50k–$75k (HR = 0.83, 95% CI: 0.74–0.94, *p* = 0.003), $75k–$100k (HR = 0.58, 95% CI: 0.50–0.69, *p* < 0.001), $100k–$150k (HR = 0.56, 95% CI: 0.47–0.65, *p* < 0.001), and >$150k (HR = 0.47, 95% CI: 0.39–0.58, *p* < 0.001) showed progressively lower risk, indicating moderate-to-large protective effects at higher income levels. The $35k–$50k income category was not statistically significant, and Unknown income was not associated with T2DM risk in adjusted models.

**Table 2:** Adjusted hazard ratios for incident type 2 diabetes mellitus after depression diagnosis (*N* = 40,585)

Variable	HR	95% CI (Lower – Upper)	*p*-value
**Gender**			
Male (vs. Female)	1.38	1.27–1.49	< 0.001 ***
Other/Unknown (vs. Female)	1.00	0.77–1.30	0.988
**Race**			
Black (vs. White)	1.79	1.64–1.95	< 0.001 ***
Asian (vs. White)	1.21	0.81–1.79	0.356
Other (vs. White)	1.25	1.09–1.44	0.002 **
Unknown (vs. White)	1.74	1.38–2.20	< 0.001 ***
**Ethnicity**			
Not Hispanic or Latino (vs. Hispanic)	1.37	1.10–1.71	0.006 **
Other/Unknown (vs. Hispanic)	1.09	0.88–1.35	0.416
**Income**			
$35k–$50k (vs. <$35k)	0.90	0.79–1.02	0.089
$50k–$75k (vs. <$35k)	0.83	0.74–0.94	0.003 **
$75k–$100k (vs. <$35k)	0.58	0.50–0.69	< 0.001 ***
$100k–$150k (vs. <$35k)	0.56	0.47–0.65	< 0.001 ***
>$150k (vs. <$35k)	0.47	0.39–0.58	< 0.001 ***
Unknown (vs. <$35k)	0.97	0.89–1.07	0.575
**Continuous Variables**			
Age (per year)	1.03	1.027–1.032	< 0.001 ***
BMI (per 10 kg/m²)	1.76	1.69–1.83	< 0.001 ***

Adjusted hazard ratios (HRs) and 95% confidence intervals (CIs) for incident type 2 diabetes mellitus (T2DM) following a depression diagnosis among 40,585 adults in the All of Us Research Program. The Cox proportional hazards model included gender, race, ethnicity, age (per year), body mass index (BMI, per 10 kg/m²), and income category. Reference groups: Female (gender), White (race), Hispanic or Latino (ethnicity), and <$35k (income). HR > 1 indicates increased risk. Statistically significant values are bolded. Model concordance = 0.711

### Interaction and stratified analyses

To assess potential modification of the BMI effect by gender, we included a BMI × gender interaction term in the model. The interaction between BMI and male gender was statistically significant (HR for Male × BMI = 1.11, 95% CI: 1.01–1.22, *p* = 0.033), indicating that the association between BMI and T2DM risk was modestly stronger among males compared to females. The interaction for Other/Unknown gender was not statistically significant (HR = 1.05, 95% CI: 0.79–1.39, *p* = 0.755).

In stratified analyses, the association between BMI and T2DM remained robust among males (HR = 1.90 per 10 kg/m², 95% CI: 1.74–2.07, *p* < 0.001), consistent with a moderate-to-large effect and somewhat stronger than that observed in the overall sample.

**Fig. 2:**
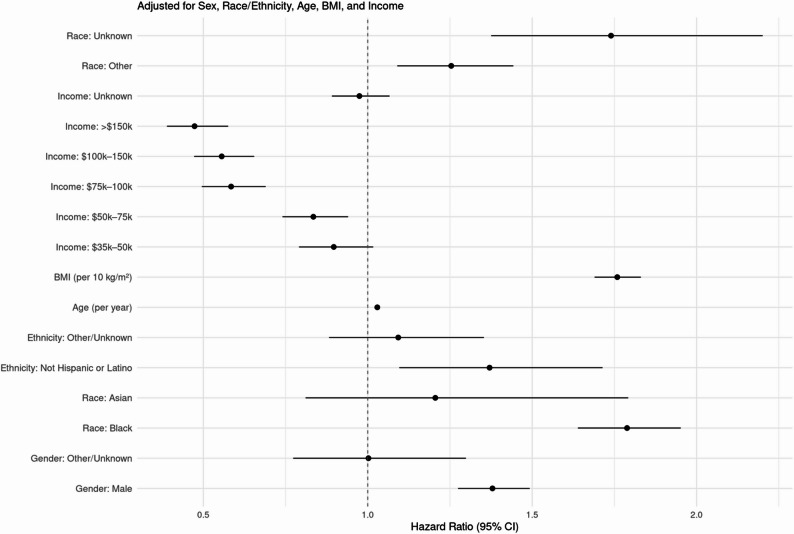
Hazard ratios for incident type 2 diabetes mellitus (T2DM) After depression diagnosis. Forest plot displaying adjusted hazard ratios (HRs) and 95% confidence intervals (CIs) for incident T2DM among 40,585 participants with a prior depression diagnosis in the All of Us Research Program. The Cox proportional hazards model estimates the relative risk of developing T2DM after depression diagnosis, as a function of baseline covariates: gender, race, ethnicity, age (per year), body mass index (BMI, per 10 kg/m²), and annual income category. Reference groups: Female (gender), White (race), Hispanic or Latino (ethnicity), and income <$35,000. Horizontal lines represent 95% CIs. HRs greater than 1 indicate increased risk of T2DM

### Smoking sensitivity analysis (Supplementary Tables 2 and 3)

To assess the robustness of our findings, we conducted a sensitivity analysis incorporating self-reported ever-smoking status, defined as endorsement of smoking ≥ 100 cigarettes lifetime. Among 40,812 participants with available smoking data, 40.3% were classified as smokers, 57.0% as non-smokers, and 2.7% had missing smoking status.

In the fully adjusted Cox model including smoking (*n* = 39,479; 3,201 events), smoking was independently associated with incident T2DM (HR = 1.12; 95% CI: 1.05–1.21; *p* = 0.001). However, inclusion of smoking produced only minimal changes in the magnitude and direction of the primary associations. BMI remained strongly associated with incident T2DM (HR = 1.76; 95% CI: 1.69–1.83), as did older age (HR = 1.03 per year; 95% CI: 1.026–1.031) and male gender (HR = 1.37; 95% CI: 1.26–1.49). Black race remained associated with substantially elevated risk (HR = 1.80; 95% CI: 1.65–1.97), and higher income categories continued to demonstrate protective associations comparable to the primary model.

Comparison with the base model excluding smoking showed only trivial shifts in effect estimates (e.g., Black race HR 1.79 vs. 1.80; male gender HR 1.38 vs. 1.37), indicating that smoking did not meaningfully confound the observed relationships. These findings support the robustness of the primary results to adjustment for smoking history.

### Missing data sensitivity analyses and multiple imputation

To assess potential bias due to missing data, we conducted a sensitivity analysis comparing individuals with complete data (*n* = 40,585) to those with missingness on key model variables (*n* = 227). As shown in **S**upplementary Table 4, individuals with missing data were more likely to be Black (27.3% vs. 16.6%) or classified as Other race (14.1% vs. 8.6%), and substantially more likely to fall into the lowest income bracket (<$35k; 63.0% vs. 37.3%), with a SMD of 0.608 for income, indicating meaningful imbalance. Participants with missing data were also younger on average (mean age 40.6 vs. 47.1 years; SMD = 0.458) and showed modest differences in sex distribution (SMD = 0.257) and race overall (SMD = 0.362). Ethnicity did not differ meaningfully between groups (SMD = 0.022). Notably, the proportion of excluded participants was small (0.6% of the analytic sample), limiting the potential impact of missing data on primary hazard ratio estimates.

To evaluate the robustness of our findings to missing BMI values, we performed multiple imputation using predictive mean matching (m = 5). The Cox proportional hazards model was re-estimated across imputed datasets and pooled. As presented in Supplementary Fig. 1, the pattern of associations remained largely consistent with the complete case model. Black race, male gender, older age, and higher BMI (per 10 kg/m²) continued to be associated with elevated risk of incident T2DM. Higher income categories remained protective in a graded fashion. Notably, the effect size estimates and confidence intervals for income, BMI, and demographic covariates were similar in direction and magnitude compared to the complete case model. Detailed pooled estimates, standard errors, and p-values are reported in Supplementary Table 5, supporting the stability of our results under assumptions of missing at random.

## Discussion

In this large, diverse cohort of adults with a diagnosis of depression, we found that greater BMI, older age, male sex, Black race, and lower household income were all independently associated with increased risk of developing T2DM. These findings identify a recognizable risk factor profile for diabetes among adults with depression, with demographic, socioeconomic, and clinical determinants showing directions of association consistent with those reported in general-population studies. Our results extend prior literature linking depression to metabolic risk, highlighting the compounding influence of sociodemographic factors and obesity on diabetes onset following depression [15, 27]. Compared with nationally representative estimates from the 2024 National Health Interview Survey [19], the All of Us depression cohort was more female, more racially diverse, and had a higher mean BMI, while mean age was similar. These differences likely reflect volunteer participation and healthcare engagement patterns inherent to large research cohorts. Nonetheless, the observation of established diabetes risk gradients within this diverse depression cohort supports the clinical relevance of these associations.

The observed association between higher BMI and incident T2DM aligns with the well-established role of adiposity in insulin resistance and metabolic dysregulation. We additionally observed a modest but statistically significant interaction between BMI and male sex, indicating that the association between adiposity and incident T2DM was slightly stronger among males than females. Although the magnitude of this interaction was small, it suggests potential sex-specific differences in metabolic vulnerability in line with existing literature [15, 28] that warrant further investigation. Notably, even after accounting for BMI, individuals identifying as Black had nearly double the risk of incident T2DM compared to White participants. This is consistent with prior findings in the general population [29], demonstrating that structural and systemic factors contributing to racial disparities in diabetes risk are equally evident among individuals with depression [12]. Our findings also echo national surveillance data showing higher diabetes burden in males than females, and in older age groups, underscoring the need for proactive screening and prevention strategies for these demographic subgroups.

Importantly, we found a robust income gradient in T2DM risk. Participants with household incomes above $75,000 had substantially lower risk, with those in the highest income bracket (>$150,000) exhibiting a 50% reduction in diabetes hazard compared to those earning less than $35,000. This social gradient has been extensively reported in general population studies [10–12, 30], but has rarely been quantified in the context of individuals with depression [31]. Our findings suggest that socioeconomic disadvantage may exacerbate diabetes vulnerability following depression much as it does in the broader population, potentially via mechanisms related to chronic stress, dietary patterns, access to care, or health literacy [10].

Smoking is a well-established risk factor for cardiometabolic disease, including T2DM [7], and was independently associated with incident diabetes in our sensitivity analysis. Participants reporting ≥ 100 lifetime cigarettes had a modest but statistically significant increased risk of T2DM after adjustment for sociodemographic and clinical covariates. Importantly, inclusion of smoking did not meaningfully alter the magnitude or direction of associations for BMI, age, gender, race, or income, indicating that smoking did not substantially confound the primary relationships of interest. The relatively modest effect size may reflect the use of a binary ever-smoking measure, which does not capture smoking intensity, duration, or recency, factors known to influence metabolic risk.

Our missing data sensitivity analyses affirmed the robustness of our findings. When we conducted multiple imputation to address a small number of missing BMI values, results were nearly identical to those of the complete-case model. This reinforces that our findings are unlikely to be artifacts of missing data. Additionally, individuals who declined to report income were retained as an informative category and not treated as missing. While these responses may reflect complex socioeconomic realities not captured by income brackets, they remained analytically distinct from true missingness.

### Limitations

Several limitations should be acknowledged. First, depression and T2DM diagnoses were identified through electronic health records, which may introduce misclassification or underdiagnosis, particularly among individuals with lower healthcare utilization. Because T2DM diagnoses were ascertained through EHR data, differential healthcare utilization could also influence detection of incident diabetes. Individuals with more frequent clinical encounters may have greater opportunity for laboratory testing and diagnostic coding. Although All of Us participants are generally healthcare-engaged, we did not explicitly adjust for visit frequency, and residual detection bias cannot be excluded. Although we restricted the depression phenotype to SNOMED CT codes for major depressive disorder and recurrent major depression to reduce heterogeneity, variation in severity and chronicity within these diagnoses remains possible. Symptom severity measures such as PHQ-9 scores were not consistently available across the cohort and were incompletely linked temporally to diagnosis dates, limiting our ability to conduct severity-stratified analyses. Additionally, although incident T2DM was defined as occurring after the index depression diagnosis, sparse and irregular longitudinal capture within the EHR limited our ability to implement fixed lag periods to further exclude potentially prevalent but previously undocumented cases. Second, although we adjusted for key clinical and demographic covariates, residual confounding by unmeasured factors remains possible. In particular, antidepressant medication use was not explicitly modeled. Certain antidepressants are associated with weight gain and metabolic changes, and prescribing patterns may vary by race, income, and healthcare setting. Within our causal framework, antidepressant use is more plausibly conceptualized as a mediator of the relationship between depression and T2DM rather than a baseline confounder; however, differential treatment patterns could contribute to observed demographic gradients. Medication data within the All of Us Research Program are heterogeneous and incompletely captured across sources, limiting reliable time-varying exposure modeling and raising concern for misclassification. Third, income was self-reported in broad categories; while true missingness was rare, a sizable proportion of participants selected “Unknown,” which may reflect diverse financial circumstances or reluctance to disclose. Fourth, covariates such as BMI and depression were treated as static and assessed at a single time point; changes over time, including weight fluctuations or recurrence of depressive episodes, were not modeled and may have influenced observed associations. Lastly, the observational design precludes causal inference.

## Conclusion

Among adults with depression, established demographic, socioeconomic, and clinical risk factors—including obesity, older age, male sex, Black race, and lower household income—were independently associated with incident type 2 diabetes mellitus. These findings quantify the magnitude and direction of traditional diabetes risk factors within a depressed cohort and underscore the continued influence of adiposity and social disadvantage on metabolic vulnerability in this population. Clinically, this suggests that individuals with depression exhibit recognizable and measurable metabolic risk gradients, reinforcing the importance of systematic diabetes screening and risk stratification within psychiatric care settings, particularly for patients with obesity or socioeconomic disadvantage. Although these associations are directionally consistent with prior literature in general populations, this study did not include a non-depressed comparator group and therefore cannot determine whether depression modifies these risk relationships. Future studies incorporating formal interaction analyses and direct comparisons by depression status are needed to clarify whether the relative strength of established diabetes risk factors differs in psychiatric versus non-psychiatric populations.

## Supplementary Information

Below is the link to the electronic supplementary material.

Supplementary Material 1

## Data Availability

The dataset supporting the conclusions of this article is available in the All of Us Research Program Registered Tier dataset (version 8) repository, accessible via the All of Us Researcher Workbench (https://www.researchallofus.org). Access to these data is restricted to qualified researchers who complete the required training and agree to the All of Us Data and Statistics Dissemination Policy. Due to participant privacy protections, the dataset is not publicly available; however, approved users may obtain access through the Researcher Workbench platform.
