# Supplementary material for: Established risk factors for type 2 diabetes in adults with depression: implications for endocrine practice
Source: BMC Endocr Disord. 2026 Jun 9;26:237. doi: 10.1186/s12902-026-02342-7 (PMC13474707; doi:10.1186/s12902-026-02342-7)
Supplement: Supplementary file 1 [file 12902_2026_2342_MOESM1_ESM.docx]

**Supplementary Appendix A. Depression Case Definition (SNOMED Concept Codes)**

Depression diagnoses were identified using standard SNOMED condition codes within the All of Us Research Program electronic health record data. To minimize diagnostic heterogeneity, the exposure definition was restricted to major depressive disorder phenotypes and did not include dysthymia, adjustment disorders with depressed mood, or other depressive-spectrum diagnoses.

Specifically, depression was defined using the following SNOMED concepts mapped within the OMOP common data model: Major depressive disorder (SNOMED CT 370143000; All of Us Concept ID 4152280) and Recurrent major depression (SNOMED CT 66344007; All of Us Concept ID 4282316). Participants were classified as having depression if at least one qualifying diagnosis code was recorded in the electronic health record prior to baseline follow-up.

**Supplementary Appendix B. Type 2 Diabetes Mellitus Case Definition (SNOMED Concept Codes)**

Incident type 2 diabetes mellitus (T2DM) was identified using standard SNOMED CT condition codes within the All of Us electronic health record data mapped to the OMOP common data model. The outcome definition was restricted to Type 2 diabetes mellitus (SNOMED CT 44054006; All of Us Concept ID 201826) and Type 2 diabetes mellitus without complication (SNOMED CT 313436004; All of Us Concept ID 4193704). Only diagnoses occurring after the index depression diagnosis were considered incident cases.

**Supplementary Table 1: Comparison of Adults with Depression: All of Us vs NHIS 2024**

| **Characteristic** | **All of Us (%) or Mean (SD)** | **NHIS 2024 (%) or Weighted Mean (SE)** |
| --- | --- | --- |
| **Sample Size** | 40,585 | 6,367 (unweighted) |
| **Age, years** | 47.08 (16.23) | 46.07 (0.30) |
| **BMI, kg/m²** | 30.8† | 28.71 (0.11) |
| **Sex (%)** |  |  |
| Female | 74.7 | 65.7 |
| Male | 23.1 | 34.2 |
| Other/Unknown | 2.2 | 0.1 |
| **Race (%)** |  |  |
| White | 55.9 | 78.6 |
| Black | 16.6 | 9.5 |
| Asian | 1.3 | 1.4 |
| Other | 8.6 | 6.7 |
| Unknown | 17.6 | 3.9 |
| **Ethnicity (%)** |  |  |
| Hispanic or Latino | 19.1 | 12.4 |
| Not Hispanic or Latino | 78.0 | 71.5 |
| Other/Unknown | 2.9 | 16.1 |

Comparison of sociodemographic characteristics among adults with depression in the All of Us Research Program and the 2024 National Health Interview Survey (NHIS). The All of Us depression cohort includes participants meeting study-specific criteria (N = 40,585). NHIS estimates are restricted to Sample Adult respondents reporting ever being told they had depression (unweighted N = 6,367) and are weighted to represent the U.S. civilian, noninstitutionalized population using survey design weights. Continuous variables are presented as mean (standard deviation) for All of Us and weighted mean (standard error) for NHIS. Categorical variables are presented as percentages. Income and smoking variables were not included due to differences in measurement and harmonization across datasets.

**Supplementary Table 2. Participant Characteristics Stratified by Incident T2DM Status Among Those with Smoking Data Available**

| **Characteristic** | **Overall** | **No T2DM** | **Incident T2DM** | **p-value** | **SMD** |
| --- | --- | --- | --- | --- | --- |
| **Sample Size** | 40,812 | 37,493 | 3,319 |  |  |
| **Gender, n (%)** |  |  |  | <0.001 | 0.096 |
| Female | 30,500 (74.7%) | 28,135 (75.0%) | 2,365 (71.3%) |  |  |
| Male | 9,418 (23.1%) | 8,527 (22.7%) | 891 (26.8%) |  |  |
| Other/Unknown | 894 (2.2%) | 831 (2.2%) | 63 (1.9%) |  |  |
| **Race, n (%)** |  |  |  | <0.001 | 0.296 |
| White | 22,781 (55.8%) | 21,251 (56.7%) | 1,530 (46.1%) |  |  |
| Black | 6,806 (16.7%) | 5,905 (15.7%) | 901 (27.1%) |  |  |
| Asian | 538 (1.3%) | 512 (1.4%) | - |  |  |
| Other | 3,516 (8.6%) | 3,241 (8.6%) | 275 (8.3%) |  |  |
| Unknown | 7,171 (17.6%) | 6,584 (17.6%) | 587 (17.7%) |  |  |
| **Ethnicity, n (%)** |  |  |  | 0.028 | 0.049 |
| Hispanic or Latino | 7,816 (19.2%) | 7,234 (19.3%) | 582 (17.5%) |  |  |
| Not Hispanic or Latino | 31,820 (78.0%) | 29,190 (77.9%) | 2,630 (79.2%) |  |  |
| Other/Unknown | 1,176 (2.9%) | 1,069 (2.9%) | 107 (3.2%) |  |  |
| **Income, n (%)** |  |  |  | <0.001 | 0.304 |
| <$35k | 15,291 (37.5%) | 13,801 (36.8%) | 1,490 (44.9%) |  |  |
| $35k–50k | 3,562 (8.7%) | 3,261 (8.7%) | 301 (9.1%) |  |  |
| $50k–75k | 4,388 (10.8%) | 4,037 (10.8%) | 351 (10.6%) |  |  |
| $75k–100k | 3,007 (7.4%) | 2,844 (7.6%) | 163 (4.9%) |  |  |
| $100k–150k | 3,548 (8.7%) | 3,381 (9.0%) | 167 (5.0%) |  |  |
| >$150k | 3,102 (7.6%) | 2,988 (8.0%) | 114 (3.4%) |  |  |
| Unknown | 7,913 (19.4%) | 7,180 (19.2%) | 733 (22.1%) |  |  |
| **Age (years), mean (SD)** | 47.04 (16.22) | 46.67 (16.43) | 51.32 (12.84) | <0.001 | 0.316 |
| **BMI (per 10 kg/m²), mean (SD)** | 3.08 (0.79) | 3.05 (0.78) | 3.45 (0.79) | <0.001 | 0.515 |
| **Smoker, n (%)** |  |  |  | <0.001 | 0.120 |
| Non-Smoker | 23,262 (57.0%) | 21,544 (57.5%) | 1,718 (51.8%) |  |  |
| Smoker | 16,440 (40.3%) | 14,922 (39.8%) | 1,518 (45.7%) |  |  |
| Missing | 1,110 (2.7%) | 1,027 (2.7%) | 83 (2.5%) |  |  |

This table presents sociodemographic and clinical characteristics for participants included in the smoking sensitivity analysis (N = 40,812), stratified by incident type 2 diabetes mellitus (T2DM) status. Smoking status was defined as endorsement of ≥100 lifetime cigarettes. Categorical variables are reported as count (percentage), and continuous variables as mean (standard deviation). P-values were calculated using chi-squared tests for categorical variables and t-tests for continuous variables. Standardized mean differences (SMDs) >0.1 indicate meaningful imbalance. Cells with counts ≤20 are masked (“-”) per All of Us policy.

**Supplementary Table 3. Adjusted Hazard Ratios for Incident Type 2 Diabetes Mellitus Including Smoking Status**

| **Variable** | **HR** | **95% CI (Lower – Upper)** | **p-value** |
| --- | --- | --- | --- |
| **Gender** |  |  |  |
| Male (vs. Female) | 1.37 | 1.26 – 1.49 | <0.001 *** |
| Other/Unknown (vs. Female) | 0.99 | 0.75 – 1.29 | 0.915 |
| **Race** |  |  |  |
| Black (vs. White) | 1.80 | 1.65 – 1.97 | <0.001 *** |
| Asian (vs. White) | 1.23 | 0.82 – 1.84 | 0.319 |
| Other (vs. White) | 1.22 | 1.05 – 1.40 | 0.008 ** |
| Unknown (vs. White) | 1.79 | 1.41 – 2.28 | <0.001 *** |
| **Ethnicity** |  |  |  |
| Not Hispanic or Latino (vs. Hispanic) | 1.39 | 1.11 – 1.75 | 0.005 ** |
| Other/Unknown (vs. Hispanic) | 1.09 | 0.88 – 1.36 | 0.438 |
| **Income** |  |  |  |
| $35k–50k (vs. <$35k) | 0.92 | 0.81 – 1.04 | 0.200 |
| $50k–75k (vs. <$35k) | 0.84 | 0.75 – 0.95 | 0.005 ** |
| $75k–100k (vs. <$35k) | 0.59 | 0.50 – 0.70 | <0.001 *** |
| $100k–150k (vs. <$35k) | 0.57 | 0.48 – 0.67 | <0.001 *** |
| >$150k (vs. <$35k) | 0.49 | 0.40 – 0.59 | <0.001 *** |
| Unknown (vs. <$35k) | 0.99 | 0.90 – 1.09 | 0.829 |
| **Continuous Variables** |  |  |  |
| Age (per year) | 1.03 | 1.026 – 1.031 | <0.001 *** |
| BMI (per 10 kg/m²) | 1.76 | 1.69 – 1.83 | <0.001 *** |
| **Smoking** |  |  |  |
| Smoker (vs. Non-Smoker) | 1.12 | 1.05 – 1.21 | 0.001 ** |

Results from a multivariable Cox proportional hazards model including smoking status as a covariate among 39,479 adults with complete data (3,201 incident events). Smoking status was defined as endorsement of ≥100 lifetime cigarettes. The model adjusts for gender, race, ethnicity, age (per year), BMI (per 10 kg/m²), income category, and smoking status. Reference groups: Female (gender), White (race), Hispanic or Latino (ethnicity), <$35k (income), and Non-Smoker (smoking status). Hazard ratios (HRs) >1 indicate increased risk of developing type 2 diabetes mellitus (T2DM). Statistical significance is denoted as follows: * p < 0.05, ** p < 0.01, *** p < 0.001. Model concordance = 0.711.

**Supplementary Table 4: Comparison of Complete-Case and Incomplete-Case Participants Used in Cox Proportional Hazards Models**

| **Characteristic** | **Overall (N=40,812)** | **Complete Case (N=40,585)** | **Missing Data (N=227)** | **p-value** | **SMD** |
| --- | --- | --- | --- | --- | --- |
| **Sex, n (%)** |  |  |  | 0.001 | 0.257 |
| Female | 30,500 (74.7%) | 30,315 (74.7%) | 185 (81.5%) |  |  |
| Male | 9,418 (23.1%) | 9,386 (23.1%) | 32 (14.1%) |  |  |
| Other/Unknown | 894 (2.2%) | 884 (2.2%) | —* |  |  |
| **Race, n (%)** |  |  |  | <0.001 | 0.362 |
| White | 22,781 (55.8%) | 22,685 (55.9%) | 96 (42.3%) |  |  |
| Black | 6,806 (16.7%) | 6,744 (16.6%) | 62 (27.3%) |  |  |
| Asian | 538 (1.3%) | 537 (1.3%) | —* |  |  |
| Other | 3,516 (8.6%) | 3,484 (8.6%) | 32 (14.1%) |  |  |
| Unknown | 7,171 (17.6%) | 7,135 (17.6%) | 36 (15.9%) |  |  |
| **Ethnicity, n (%)** |  |  |  | 0.949 | 0.022 |
| Hispanic or Latino | 7,816 (19.2%) | 7,771 (19.1%) | 45 (19.8%) |  |  |
| Not Hispanic or Latino | 31,820 (78.0%) | 31,644 (78.0%) | 176 (77.5%) |  |  |
| Other/Unknown | 1,176 (2.9%) | 1,170 (2.9%) | —* |  |  |
| **Income, n (%)** |  |  |  | <0.001 | 0.608 |
| <$35k | 15,291 (37.5%) | 15,148 (37.3%) | 143 (63.0%) |  |  |
| $35k–50k | 3,562 (8.7%) | 3,542 (8.7%) | —* |  |  |
| $50k–75k | 4,388 (10.8%) | 4,371 (10.8%) | —* |  |  |
| $75k–100k | 3,007 (7.4%) | 2,999 (7.4%) | —* |  |  |
| $100k–150k | 3,548 (8.7%) | 3,540 (8.7%) | —* |  |  |
| >$150k | 3,102 (7.6%) | 3,098 (7.6%) | —* |  |  |
| Unknown | 7,913 (19.4%) | 7,887 (19.4%) | 26 (11.5%) |  |  |
| **Age, mean (SD)** | 47.04 (16.22) | 47.08 (16.23) | 40.55 (11.98) | <0.001 | 0.458 |
| **BMI (per 10 kg/m²), mean (SD)** | 3.08 (0.79) | 3.08 (0.79) | —* | —* | —* |

Comparison of participants included in the complete-case Cox proportional hazards analysis versus those excluded due to missing covariate data. Categorical variables are presented as count (percentage), and continuous variables as mean (standard deviation). P-values were calculated using chi-squared tests for categorical variables and t-tests for continuous variables where estimable. Standardized mean differences (SMDs) are shown to quantify imbalance; values >0.1 indicate meaningful differences between groups. The number of participants excluded due to missing data was small (N=227; 0.6% of the analytic sample). Although excluded participants differed in age, race, sex distribution, and income, the limited proportion of excluded individuals (0.6%) suggests minimal impact on primary hazard ratio estimates.

*Value suppressed due to cell size ≤20 per All of Us data dissemination policy.

**Supplementary Table 5: Pooled Hazard Ratios for Incident T2DM After Depression Diagnosis**

| **Variable** | **HR** | **95% CI (Lower – Upper)** | **p-value** |
| --- | --- | --- | --- |
| **Gender** |  |  |  |
| Male (vs. Female) | 1.37 | 1.27 – 1.49 | <0.001 *** |
| Other/Unknown (vs. Female) | 1.05 | 0.82 – 1.34 | 0.692 |
| **Race** |  |  |  |
| Black (vs. White) | 1.78 | 1.63 – 1.95 | <0.001 *** |
| Asian (vs. White) | 1.24 | 0.84 – 1.83 | 0.276 |
| Other (vs. White) | 1.27 | 1.10 – 1.46 | <0.001 *** |
| Unknown (vs. White) | 1.70 | 1.35 – 2.14 | <0.001 *** |
| **Ethnicity** |  |  |  |
| Not Hispanic or Latino (vs. Hispanic) | 1.34 | 1.08 – 1.66 | 0.008 ** |
| Other/Unknown (vs. Hispanic) | 1.09 | 0.88 – 1.36 | 0.408 |
| **Continuous Variables** |  |  |  |
| Age (per year) | 1.03 | 1.026 – 1.031 | <0.001 *** |
| BMI (per 10 kg/m²) | 1.75 | 1.68 – 1.82 | <0.001 *** |
| **Income** |  |  |  |
| $35k–50k (vs. <$35k) | 0.89 | 0.79 – 1.01 | 0.069 |
| $50k–75k (vs. <$35k) | 0.83 | 0.74 – 0.94 | 0.003 ** |
| $75k–100k (vs. <$35k) | 0.59 | 0.50 – 0.70 | <0.001 *** |
| $100k–150k (vs. <$35k) | 0.55 | 0.47 – 0.65 | <0.001 *** |
| >$150k (vs. <$35k) | 0.47 | 0.39 – 0.57 | <0.001 *** |
| Unknown (vs. <$35k) | 0.97 | 0.89 – 1.07 | 0.458 |

Pooled hazard ratios (HRs), 95% confidence intervals (CIs), and p-values from a multivariable Cox proportional hazards model predicting incident type 2 diabetes mellitus (T2DM) following a diagnosis of depression in the All of Us cohort. BMI values were multiply imputed using predictive mean matching (m = 5). The model includes gender, race, ethnicity, age (per year), BMI (per 10 kg/m²), and income category as covariates. Reference groups: Female (gender), White (race), Hispanic or Latino (ethnicity), and <$35k (income). HRs > 1 indicate increased risk of T2DM.

**Supplementary Figure 1. Pooled Hazard Ratios for Incident T2DM After Depression Diagnosis (BMI Imputed)**

Forest plot showing pooled hazard ratios (HRs) and 95% confidence intervals (CIs) from a multivariable Cox proportional hazards model predicting incident type 2 diabetes mellitus (T2DM) following a diagnosis of depression. Model covariates included gender, race, ethnicity, age (per year), body mass index (BMI, per 10 kg/m²), and income category. Missing BMI values were multiply imputed using predictive mean matching (m = 5). Reference groups: Female (gender), White (race), and Hispanic or Latino (ethnicity). Horizontal bars represent 95% confidence intervals. HRs > 1 indicate increased risk of T2DM.
